# The Effects of Mid-life Stress Exposure on Black-White Differences in Cognitive Decline

**DOI:** 10.1093/geroni/igab046.372

**Published:** 2021-12-17

**Authors:** Uchechi Mitchell

**Affiliations:** University of Illinois Chicago, School of Public Health, Chicago, Illinois, United States

## Abstract

Cognitive decline is a precursor to cognitive impairment and dementia. Recent research suggests that cognitive decline may begin earlier in the life course for Blacks and that Black-white disparities in cognitive function emerge in midlife. Disproportionate exposure to chronic and acute stressors during mid-life may explain Black-white differences in trajectories of cognitive function over time. In this study we use data from approximately 3,700 Black and white respondents age 51-64 from the Health and Retirement Study to examine race differences in cognitive decline and the role mid-life stress exposure play in these differences. Initial findings suggest that mid-life Blacks have lower levels of cognitive function, but their rates of cognitive decline do not differ significantly from mid-life whites. Financial strain and everyday experiences of discrimination are inversely associated with cognitive decline and only partially explain differences in cognitive decline between mid-life Blacks and whites.

